# The educational value of sense of coherence for grief care

**DOI:** 10.3389/fpsyg.2022.1037637

**Published:** 2022-11-07

**Authors:** Shisei Tei, Junya Fujino

**Affiliations:** ^1^Department of Psychiatry, Kyoto University, Kyoto, Japan; ^2^Institute of Applied Brain Sciences, Waseda University, Saitama, Japan; ^3^School of Human and Social Sciences, Tokyo International University, Saitama, Japan; ^4^Medical Institute of Developmental Disabilities Research, Showa University, Tokyo, Japan; ^5^Department of Psychiatry and Behavioral Sciences, Tokyo Medical and Dental University, Tokyo, Japan

**Keywords:** resilience, psychological, adaptation, grief, prolonged grief disorder, medical education

## Introduction

Coping and managing grief are commonplace in clinical practice but have become more pervasive issues due to the COVID-19 pandemic, recent wars, and other disasters (Wallace et al., 2020; Mitima-Verloop et al., 2022). Grief experiences have largely increased due to unexpected deaths, unnatural losses, social isolation, survivor guilt (e.g., thoughts that the mourner was the source of death or infection of the deceased), and disenfranchisement (feeling of loss that is unrecognized by others and a sense of inability to support the dying person; Amy and Doka, 2021). In this relation, complicated grief (CG) has rapidly emerged, including experiences of having recurrent intrusive thoughts about a person who died, being preoccupied with sorrow, and perceiving life as being purposeless.

Pathological departures from normal grief are on the rise and stem from a failure to mourn appropriately. During the COVID-19 pandemic, an estimated 50% of those facing bereavement required mental healthcare (Harrop et al., 2021). Furthermore, 10% of bereaved people may be diagnosed as having prolonged grief disorder (PGD), that is, a pervasive preoccupation with thoughts or memories of the deceased person for at least 12-months, with intense emotional pain or distress and substantially impaired functioning (Prigerson et al., 2021).

After nearly two decades of debates, PGD criteria are currently included in the Diagnostic and Statistical Manual of Mental Disorders (DSM-5-TR) (Prigerson et al., 2021). Therefore, more knowledge of CG and PGD are essential. Accordingly, medical professionals must necessarily develop their grief management skills (Wallace et al., 2020), thereby helping patients and their families by providing grief support.

Despite the heightened morbidity associated with grief, it receives insufficient coverage in medical education. According to some scholars in this field, instruction on grief care and end-of-life communication are still inadequate, and educational tools for bereavement require further development (e.g., Serwint et al., 2016; Baran and Justin, 2019). Grief care and coping skills are challenging to teach as they require an experience-induced empathic approach toward the bereaved (Sikstrom et al., 2019). Moreover, when facing a patient's sudden death or suicide, medical professionals may face difficulty in dealing with not only the grief of the deceased's family and friends but also their own; and grief experience can also develop secondary traumatic stress and/or empathic distress (Tei et al., 2014). Grief management aims to improve the wellness of medical professionals, patients, and patients' families. Hence, developing an educational curriculum that targets these aspects of medicine is critical. However, there is limited evidence regarding the impact of the existing curricular interventions or conventional grief education (Boland et al., 2019; Sikstrom et al., 2019).

## Current advances in teaching grief care

Some teaching materials can be taught didactically, yet others need to be conveyed in different formats. Besides the typical classroom teaching and case studies, additional approaches have been developed to complement the traditional educational curricula (Serwint et al., 2016). For example, workshops including reflective writing exercises and role-play have been organized in some universities to integrate theory and practice in simulation (Sikstrom et al., 2019; Hatlevik and Hovdenak, 2020).

### Film-based learning

Film-based learning is a powerful teaching and therapeutic tool to manage grief and develop empathy for grieving (Lumlertgul et al., 2009). This approach allows the introduction of the psychological and social complexities of grief. Viewers can empathize with film characters and reflect on various experiences of suffering, grieving process, and different ways of overcoming grief. Analyzing the film brings a unique opportunity to confront behaviors of individuals who are complex, challenging, and whom we do not meet frequently in practice. This might help learners to grasp and practice grief management skills more effectively (Darbyshire and Baker, 2012). In general, the first step in the film-based approach is to choose movies suitable for introducing the target concepts (Webster et al., 2015). The next step is to clarify the relevant experience featured in these films more rigorously. Subsequently, the workshop is usually organized so that it starts with introducing the target concepts, followed by film projection.

### Sense of coherence (SOC)

Grief can be alleviated by one's psychological ability, the so-called resilience or SOC (Antonovsky, 1987). SOC is a flexible coping capacity that can be modified by life experiences. SOC is defined as “a global life orientation that expresses the extent to which one has a pervasive, enduring, but dynamic feeling of confidence”. Previous research suggested that the more SOC a person has, the greater they can understand (comprehensibility), handle (manageability), make sense (meaningfulness) of life experiences and cope with critical situations (Eriksson and Lindström, 2005; Tei et al., 2015). Consequently, individuals with a lower SOC may exhibit a much more maladaptive form of grief, empathy, and burnout (compassion fatigue) than those with higher SOC (Kaya-Demir and Çirakoglu, 2021).

For grieving patients and their families, medical professionals play an important role in helping them to process their feelings of loss. In this endeavor, it is crucial for medical professionals to support patients in improving their SOC and simultaneously developing their own (Tei et al., 2015; Veronese et al., 2021). Thus, SOC can become one of the most important capacities in clinical practice and/or a vital resource in a medical career (Tartas et al., 2014; Luibl et al., 2021). However, similar to empathy, SOC is difficult to teach as it requires hands-on learning to foster these skills in a heuristic manner.

Recent studies have been successful in developing SOC (Super et al., 2016; Hatlevik and Hovdenak, 2020) and support the concept that film-based experiential approaches might help in improving empathic abilities and SOC to manage grief (Lumlertgul et al., 2009; Izod and Dovalis, 2015). Fostering SOC to examine different perspectives and consider one's immediate, social, and cultural environment and engaging in critical dialogs with others may be important (Ozcakir and Bilgel, 2014). These skills can deepen a person's understanding of diverse stressful situations and increase stress coping resources, which are required to overcome grief.

This opinion article, recommended for instructors and clinicians, addresses the potential role of SOC in grief care. Moreover, we delineate a film-based approach in introducing the grieving processes and developing SOC. In this endeavor, based on previous studies (Blanco-Canitrot et al., 2018; Attwood, 2021; Sunarsi et al., 2022), we also explored complicated, prolonged, and disenfranchised grief, as well as related experiences (e.g., survivor guilt) featured in the selected film to extend this discussion. We selected films depicting a range of bereavement narratives wherein SOC helped alleviate the burden of grief. We searched for inherent SOC embedded in grievers adapting to their loss. Additionally, we investigated relevant articles (e.g., reviews of films) and performed consensus ranking to screen the films. In addition to films from the United States and Europe, we included an Asian film in an attempt to explore a broader perspective that considers cultural and societal factors as a critical component in managing grief and expanding SOC (please see Supplementary material). Consequently, we discussed the interpretation of these films with our students and faculty, based on previous studies (Ozcakir and Bilgel, 2014). In the following paragraphs, we provide a discussion on grief care and coping tools depicted by these films in light of SOC. Subsequently, a film-based approach is introduced.

According to the literature, we speculate that SOC and its film-based learning appear relevant and effective in introducing grief care. This approach might improve students' attitudes toward death and may enhance their ability to tolerate their own anxiety or distress when experiencing others' grief. Whether and how they can help impart grief management skills appears worthy of further investigation. It is possible that the approach can prompt learners to explore and discuss the concept of SOC in relation to CG and PGD.

The film-based setting may allow us to recapture the role of SOC in grief management. Indeed, the reviewed films demonstrated that dealing with grief involves efforts to support others (as well as oneself) and feel connected to humanity again *via* empathy and SOC. In other words, sharing one's loss and endeavoring to understand others' suffering may cultivate SOC that can assuage feelings of hopelessness. In the film review, the feelings of worthlessness that Hanna and Lee faced represent their altered sense of value or SOC (please see Supplementary material). Contrastingly, the sense of meaning in life and SOC that Tokue described in her life story may have counteracted her hardship and grief. People's beliefs about themselves, life, and death can have significant effects on mitigating grief and cultivating SOC through the contextualization of the loss (Jawaid et al., 2022; Tei, 2022).

The reviewed films also provide the following clues for fostering SOC; (1) facing and searching for the meaning of loss in *The Secret Life of Words* (i.e., meaningfulness factor in SOC), (2) acknowledging the past and engendering hope for the future in *Manchester by the Sea* (comprehensibility), and (3) realizing one's own ability in *Sweet Bean* (manageability). These three factors can help to perceive life as meaningful (adversities are viewed as challenges and worthy of engagement), manageable (sufficient resources to overcome hardships are felt as available), and comprehensible (rational and predictable).

This article can be utilized in teaching grief care and SOC in various ways. In a grief care workshop that we held, films and SOC were briefly introduced by instructors in the context of grief coping and management. Specifically, instructors first explained the concepts of grief care and SOC as a part of their regular curriculum at the university. Next, the attendees watched the films, and subsequently, group discussions were conducted, and feedback was received, illuminating several psychological processes of grief and SOC. The feedback shared between learners allowed them to broaden their perspectives on grief and SOC. Instructors can read this article to collect pertinent information. Then, they can stimulate group discussions with their learners and ask open-ended questions, which are tailored to their teaching goals. Instructors and learners can discuss how the provision of psychiatric input, death-related beliefs, and clinical interventions might have influenced the course of the depicted behaviors in films. This approach may be enriched through raising problems, debating, and reflecting. Self-reflection can nurture the learner's ability to deal with change, empathize with patients/families, and manage emotions after difficult patient care experiences (e.g., through flexible, context-adjusted perspective-taking; Serwint et al., 2016; Tei and Fujino, 2022). Armed with such skills, learners may be better poised to provide grief care. These skills may further support learners and patients in shifting their perspective or attention from their deceased loved ones to the world around them and to their future.

Experiences during film viewing can considerably vary among individuals (Darbyshire and Baker, 2012; Tei et al., 2020), and psychopathic behaviors described in films can easily bias the viewers; however, such biases can be appreciated because understanding our individual variances is one of the crucial aspects of clinical practice. Stimulating discussions about the learners' diversity in experiences can promote understanding of nuanced psychopathic behaviors and illuminate the importance of personalized care (Prigerson et al., 2021). Meanwhile, subsequent teaching is certainly required to avoid misunderstanding. To this end, it is crucial to realize that when facing death, how people perceive, react, understand, and make memories can differ significantly. Indeed, Karl Jaspers once mentioned that the *form* provides a firm ground for classifications of psychopathological phenomena; however, their *content* can become essential as it is inevitably influenced by individual experiences (Tei and Wu, 2021).

## Conclusions

We posited SOC as a resource that can possibly help alleviate the aggravation of bereavement and thus be a catalyst for medical students and trainees/residents to cultivate their coping skills in grief care. Future research should incorporate more empirical studies to examine the impact of the film-based approach on introducing grieving processes and developing SOC. Hopefully, this commentary will stimulate further discussions and facilitate in motivating readers to develop a more engaging pedagogy for grief care, as well as to develop SOC and competency in coping with grief, thereby providing compassionate care.

## Author contributions

ST and JF designed the work. ST wrote the first draft. Both authors contributed to the article and approved the submitted version.

## Funding

This work was supported by the Ministry of Education, Culture, Sports, Science and Technology of Japan (21K07544, 20K16654).

## Conflict of interest

The authors declare that the research was conducted in the absence of any commercial or financial relationships that could be construed as a potential conflict of interest.

## Publisher's note

All claims expressed in this article are solely those of the authors and do not necessarily represent those of their affiliated organizations, or those of the publisher, the editors and the reviewers. Any product that may be evaluated in this article, or claim that may be made by its manufacturer, is not guaranteed or endorsed by the publisher.
