# Supplementary material for: The educational value of sense of coherence for grief care
Source: Front Psychol. 2022 Nov 7;13:1037637. doi: 10.3389/fpsyg.2022.1037637 (PMC9676356; doi:10.3389/fpsyg.2022.1037637)
Supplement: Supplementary file 1 [file Table_1.DOCX]

Supplementary Material

# Supplementary Data

**Table 1. Film Review**

| **The Secret Life of Words**  Isabel Coixet’s film (2005) examines grief and survivor guilt. Hanna is a deaf, silent, reclusive young woman working in a factory. During vacation, she finds a nursing job on an oil rig in the Irish Sea that involves caring for Josef, a burn victim who has temporarily lost his sight. The two gradually become close and start sharing their suffering. Josef reveals his painful past of self-condemnation for his best friend’s suicide, possibly triggered by Josef’s misconduct. Meanwhile, Hanna gradually discloses that she is a survivor of the Balkan war and was once captured and tortured. Hanna is traumatized and filled with regret and sorrow as her closest friend died and she did not.  Viewers may acknowledge Hanna’s severe grief, such as preoccupation with self-blame, sadness, and perceived meaninglessness of her life. She frequently removes her hearing aid to withdraw from reality, and she eats nothing but chicken and rice without enjoyment. These behaviors, together with shame and stigma, can be regarded as examples of CG (Wallace et al., 2020). Viewers can also experience how suffering might be eased by continued dialog. Hanna and Josef reconcile with their pain and self-blame through their gradual acceptance of past incidents.  We may discover that not just sharing the loss experience but also attempting to explore the meaning and/or different perspectives in the loss reduce grief, and nurture SOC or resilience to uncertainty. The film ends with Josef’s discharge from the hospital and his visit to Hanna’s factory. A reference to learning swimming was used as a metaphor for dealing with obstacles and beginning a new phase of reorganizing their lives. |
| --- |
| **Manchester by the Sea**  The film, directed by Kenneth Lonergan (2016), depicts despair, prolonged disorganization over the family loss, and pangs of grief. The story focuses on a self-punishing, depressive middle-aged loner, Lee, who slowly returns to life after the brutal death of his family. Although paralyzed by grief, Lee is also trapped by guilt and anger, partly convinced that what had occurred was his fault. Consequently, he is unable to apologize or accept apologies from others.  Complicated and prolonged grief is described effectively through Lee, who expresses repeated intrusive thoughts of the loss and perceived purposelessness of life. Viewers also encounter Lee in a phase of numbing followed by a pathological phase when he physically and emotionally isolates himself from his community and his past. These attitudes are often manifested in PGD (Prigerson et al., 2021).  Finally, we may recognize how grief can be better handled by a more sympathetic conversation and affective sharing. These attitudes may cultivate a sense of meaning in life to prompt adaptable, flexible understandings of given situations (Antonovsky, 1987). When he returns to his hometown, Manchester, Lee is forced to face the tragedy; however, he gradually shares his pain with his former wife. The film ends with a scene where Lee and his nephew, Patrick, talk about their future, reflecting slight hope. |
| **Sweet Bean**  This 2015 film, directed by Naomi Kawase, captures solitude, stigma, and disenfranchised grief. Sentaro, a loner, works at a small bakery in Tokyo. One day, he hires Tokue, an old woman, to help him at the bakery after he had tasted her delicious homemade red bean paste. The business starts to succeed; however, when customers notice the deformities in Tokue’s hand caused by leprosy, they stop coming. The manager of the bakery forced Sentaro to let her go. Later, Sentaro visits Tokue at the sanatorium where she was staying under a compulsory hospitalization system. Sentaro learns that while Tokue faced significant stigma and death, she remained optimistic, discovering comfort and meaning in her life.  Besides Tokue’s disenfranchised grief, viewers may also realize Sentaro’s preoccupation with sorrow. After visiting Tokue, Sentaro writes to Tokue and shares his long-hidden grief over the loss of his mother, who had visited him frequently during his imprisonment (despite his repeated rejection) and passed away before he was discharged from the prison. Sentaro’s experience manifests signs of CG and PGD.  Furthermore, we may recognize how grief experiences can also nurture resilience and self-efficacy (i.e., manageability in SOC). Soon after Sentaro’s visit, Tokue dies and leaves him a letter describing her gratitude for finding a life purpose, and this positively influences Sentaro’s behavior. At the film’s end, Sentaro is seen selling pancakes from his own stall in the park, with cherry trees in full bloom. Although cherry blossoms are frequently used to represent death in Japan, many appreciate them as a symbol of self-reflection and a reflection on both the transience and eternity of life. |
